# Short-Term Effects of Gender-Affirming Hormone Therapy on Dysphoria and Quality of Life in Transgender Individuals: A Prospective Controlled Study

**DOI:** 10.3389/fendo.2021.717766

**Published:** 2021-07-29

**Authors:** Lucas Foster Skewis, Ingrid Bretherton, Shalem Y. Leemaqz, Jeffrey D. Zajac, Ada S. Cheung

**Affiliations:** ^1^Trans Health Research Group, Department of Medicine (Austin Health), The University of Melbourne, VIC, Australia; ^2^Department of Endocrinology, Austin Health, VIC, Australia; ^3^College of Medicine and Public Health, Flinders University, Adelaide, SA, Australia

**Keywords:** transgender, gender dysphoria, quality of life, testosterone, oestradiol

## Abstract

**Background:**

Gender affirming hormone therapy (GAHT), whilst considered the standard of care in clinical guidelines for the treatment of many transgender (trans) people is supported by low quality evidence. In this prospective longitudinal controlled study, we aimed to examine the effect of newly commencing GAHT on gender dysphoria and quality of life (QoL) over a 6 month period.

**Methods:**

Adult trans (including those with binary and/or non-binary identities) people newly commencing standard full-doses of masculinising (*n* = 42; 35 = trans masculine, 7 = non-binary) or feminising (*n* = 35; 33 = trans feminine, 2 = non-binary) GAHT and cisgender participants (*n*=53 male, *n*=50 female) were recruited to participate in this longitudinal prospective study. This analysis of gender dysphoria measured by the Gender Preoccupation and Stability Questionnaire and QoL measured by the RAND Short-Form 36 Health survey at baseline, 3 and 6 months after commencement of GAHT was a prespecified secondary outcome. Dysphoria and QoL over time in those starting GAHT compared to cisgender comparison group matched for their presumed sex at birth is reported as the mean difference (95% confidence interval) adjusted for age.

**Results:**

In trans people initiating masculinising GAHT, there was a decrease in gender dysphoria with adjusted mean difference -6.80 (-8.68, -4.91), *p < 0.001*, and a clinically significant improvement in emotional well-being [adjusted mean difference 7.48 (1.32, 13.64), *p = 0.018*] and social functioning [adjusted mean difference 12.50 (2.84, 22.15), *p = 0.011*] aspects of QoL over the first 6 months of treatment relative to the cisgender female comparison group. No significant differences were observed in other QoL domains. In trans people initiating feminising GAHT, there was a decrease in gender dysphoria [adjusted mean difference -4.22 (-6.21, -2.24), *p < 0.001*] but no differences in any aspects of QoL were observed.

**Conclusions:**

In the short-term, our findings support the benefit of initiating masculinising or feminising GAHT for gender dysphoria. Masculinising GAHT improves emotional well-being and social functioning within 6 months of treatment. Multidisciplinary input with speech pathology and surgery to support trans people seeking feminisation is likely needed. Further longitudinal studies controlled for other confounders (such as the presence of social supports) contributing to QoL are needed.

## Introduction

Transgender individuals (trans) experience a gender identity (which may be binary or non-binary) different from that which they were presumed at birth, and often, but not always, this gender incongruency is associated with significant psychological distress, known as gender dysphoria (1, 2). Despite considerable progress made by the trans community over the past decade in regard to visibility and acceptance, trans people still face significant obstacles and prejudice (3, 4), social stigma and discrimination, leading to job and housing insecurity, verbal and physical violence, and barriers to accessing appropriate healthcare (3). Similarly, trans people experience alarmingly high rates of mental health disorders compared to the general population (5, 6). A recent study looking at self-reported mental health diagnoses within the trans community has indicated that 73% of trans adults have been diagnosed with depression in their lifetime, 67% have been diagnosed with anxiety, and 43% attempted suicide in their lifetime (3). In contrast, self-reported rates of depression, anxiety and suicide attempts for the general population are indicated at 11.6%, 26.3%, and 3.2% respectively (7). While transgender health research is still in its infancy, research thus far has indicated that gender-affirming interventions (gender counselling, gender confirmation surgery and hormone therapy), as well as sociocultural factors (external validation of one’s gender, support from family and friends) are associated with improved well-being among transgender individuals (8, 9). Access to such healthcare can be challenging for some, and the largest contributing factor to this, as reported by trans individuals, is an overall lack of medical professionals who are experienced in the field (6, 9, 10). Other reported factors include: financial and socioeconomic status, and discrimination (10). Gender affirming hormone therapy (GAHT) is the current standard treatment for people who experience gender dysphoria (5). While there is a significant amount of expert-opinion based evidence available regarding the benefits of GAHT for trans people, the majority of existing research into gender dysphoria and quality of life (QoL) focuses on mental health outcomes following surgery, and there is scant clinical evidence available regarding the effects of GAHT alone (5), particularly in regard to gender dysphoria outcomes (11). Of the research that does exist, a beneficial role of GAHT has been consistently suggested (6, 11–22), however evidence quality is low, primarily consisting of uncontrolled cross-sectional and retrospective cohort studies, and only two studies specifically quantify changes in gender dysphoria following GAHT (11, 23, 24). Consequently, existing guidelines for the treatment of trans individuals contain broad recommendations based on expert-opinion which has been valuable in providing clinical care for the trans community but is also the subject of critiques from some clinicians (2, 5). This guideline ambiguity leads to varied interpretation of treatment recommendations amongst clinicians, and a lack of consistent training practices (5).

We aimed to assess the short-term effects (0-6 months) of newly commencing GAHT on the QoL and gender dysphoria experienced by trans individuals and compare these results with age-matched cisgender individuals of the same presumed sex at birth. Based on previous literature (11, 22–24), it was hypothesised that GAHT would be associated with improved QoL and reduced gender dysphoria for trans people relative to cisgender comparison groups of the same presumed sex at birth.

## Methods

### Participants

A total of 77 trans (*n* = 35 initiating feminising GAHT, *n* = 42 initiating masculinising GAHT) and 103 cisgender (*n* = 53 male, *n* = 50 female) participants were recruited from online and local print advertisements, and primary or secondary care clinics specialising in trans health in Melbourne, Australia. Cisgender females and males in the community were individuals who responded to local advertisements for a study on bone health. Recruitment occurred between April 2017 – April 2020 for the primary outcome of bone microarchitecture, and gender dysphoria and QoL were prespecified secondary outcomes. The research study is registered with the ANZ Clinical Trials Registry ACTRN12617000584336. Trans individuals were included for the study if they were aged 18 years or over, newly commencing standard (full) doses of masculinising or feminising GAHT, and were able to provide written informed consent to the study and comply with study protocols. Cisgender individuals without medical conditions or medications that contributed to metabolic bone disease were included. Trans individuals were excluded if they had any contraindications to GAHT use, had previously used masculinising or feminising GAHT, or had a history of gender-affirming surgery. Exclusion criteria for all participants were the presence of metabolic bone disease or receiving therapy that affects bone (glucocorticoids, bisphosphonates, antiepileptic medication, use of HIV pre-exposure prophylaxis). People presumed to be menopausal (age > 50 years) (trans men and cisgender females) were excluded. Baseline study visits occurred prior to or within 4 weeks of commencing GAHT. The research study is registered with the ANZ Clinical Trials Registry ACTRN12617000584336.

### Ethics

Ethics approval was received from the Austin Health Human Research Ethics Committee (Austin/17/HREC/74) and all participants provided written informed consent.

### Design

The study incorporated a prospective controlled observational study design. Trans participants newly initiating were divided into the following groups: masculinising GAHT or feminising GAHT. Comparison individuals were presumed sex at birth-matched cisgender males and females who were not undergoing hormone therapy and we had intended to match for age as closely as possible. Final statistical analyses were adjusted for age. A comparison group who were trans but not using GAHT were not recruited as trans community members deemed that it would not be ethical or acceptable to withhold GAHT for research purposes. Participants completed the following outcomes at baseline and 6 months.

### Outcomes

#### Gender Dysphoria

Gender dysphoria was assessed using the Gender Preoccupation and Stability Questionnaire (GPSQ). The GPSQ has been validated in Australia for use as a tool to measure gender dysphoria within the transgender community (25) and was chosen for this study due to its ability to evaluate the effectiveness of gender-affirming interventions in both binary and non-binary transgender individuals. The questionnaire includes 14 multiple choice questions designed to measure the extent of an individuals’ preoccupation with gender, and the stability of their own gender identity over the past 2 weeks (25). Each question had 5 possible answers, corresponding to a score of 1-5, with higher scores indicating higher levels of gender dysphoria. Total scores were calculated by taking the summation of all values. Total scores 28 were considered highly suggestive of clinical gender dysphoria, and a change in score of 11 points or more reliably indicated a change in the degree of gender dysphoria (25).

#### Quality of Life

QoL was measured using the RAND Short Form-36 (SF-36) Health Survey, a 36 item questionnaire which has been validated for use to assess 8 domains of QoL: physical functioning (relating to the extent of physical limitations), social functioning (relating to one’s ability to participate in social activities), role limitations due to physical health (relating to the extent individuals were limited in work/activities due to physical health), role limitations due to emotional health (relating to the extent individuals were limited in work/activities due to mental health), pain (relating to physical pain), energy/fatigue (relating to one’s energy levels/vitality), general health (relating to one’s perceived physical wellbeing), and emotional wellbeing (relating to perceived mental wellbeing) (26). Each item was denoted a value of 0-100, with higher scores indicating a better health state. Scores were calculated by taking the averages from the values pertaining to each QoL domain, thus producing eight different total scores between 0-100 per visit (27). The SF-36 has been reliably used to assess the QoL of individuals in various populations and circumstances (26, 28, 29), including several that have assessed the QoL of transgender individuals on GAHT (20, 21, 30). Furthermore, it is a fast, inexpensive, and accurate tool designed to be self-reported by individuals in order to monitor and assess treatment outcomes (27, 28). The minimum clinically important difference for the SF-36 is typically in the range of 3 to 5 points which translates into a 0.09-0.28 effect size range (31).

#### Statistical Analysis

Age was not normally distributed and is presented as median (interquartile range). Descriptive statistics were otherwise presented as mean standard deviation (SD for continuous variables). Results were summarised as the mean difference [95% confidence interval (CI)] between groups (transgender versus comparison group) over time. Comparison between two groups at baseline was conducted using a two-sample independent t-test whenever a variable followed normal distribution, otherwise nonparametric Mann-Whitney test was used. Categorical variables at baseline were compared using Chi-squared test, or Fisher’s exact test where cell counts are low. Linear mixed effects model was used to test the differences between groups across time, with a time by group interaction term and a random intercept for individual participant, assuming an unstructured variance-covariance structure. The interaction term coefficients were reported, which indicates the change in trend across time between groups (i.e., whether the groups follow the same pattern of change). All models were adjusted for age. All participants recruited into the study at baseline (n=180) were used in the linear mixed effects model, in which missing data were handled *via* maximum likelihood estimation. The significance level was defined as *P* < 0.05 (two-tailed).

## Results

Baseline characteristics for the trans participants and cisgender comparison group are summarised in **Table 1** for each group. The median age of the masculinising hormone therapy group was 24.0 years (22.0-29.3) and feminising hormone therapy group was 28.0 years (22.5-39.5) relative to cisgender females [27.5 years (24.0-37.5)] and cisgender males [31.0 years (25.0-41.0)] (**Table 1**).

**Table 1:** Characteristics of trans participants and cisgender comparison group matched for presumed sex at birth.

		Masculinising GAHT group	Cisgender Females	*p* value	Feminising GAHT group	Cisgender Males	*p* value
**General Characteristics**	Total Count	42	50		35	53	
	Age Median (IQR)	24.0 (22.0-29.3)	27.5 (24.0-37.5)	0.028	28.0 (22.5-39.5)	31.0 (25.0-41.0)	0.651
**Employment Status: N (%)**	*Unemployed*	4 (10.5%)	8 (17.0%)	0.010^	8 (24.2%)	2 (4.1%)	0.018^
	*Self Employed*	2 (5.3%)	1 (2.1%)		1 (3.0%)	1 (2.0%)	
	*Casual/Part-time Employment*	21 (55.3%)	15 (31.9%)		13 (39.4%)	14 (28.6%)	
	*Full Time Employment*	8 (21.1%)	21 (44.7%)		9 (27.3%)	28 (57.1%)	
	*Retired*	0 (0.0%)	1 (2.1%)		2 (6.1%)	2 (4.1%)	
	*Other*	3 (7.9%)	1 (2.1%)		0 (0.0%)	2 (4.1%)	
**Lifetime Mental Health Diagnoses: N (%)**	*Depression*	25 (59.5%)	8 (16.0%)	<0.001	16 (45.7%)	4 (7.5%)	<0.001
	*Anxiety*	24 (57.1%)	8 (16.0%)	<0.001	14 (40.0%)	3 (5.7%)	<0.001

^Global p-values have been reported for employment status and no post-hoc analyses were performed.

### Gender Dysphoria

Compared to the cisgender comparison groups, a significant reduction in gender dysphoria within the first 6 months of GAHT was observed for both the masculinising hormone [adjusted mean difference -6.80 points (-8.68, -4.91), *p* < 0.001] and feminising hormone [adjusted mean difference -4.22 points (-6.21, -2.24), *p* < 0.001] groups, as shown in **Table 2**.

**Table 2:** Overall mean differences in GPSQ and QoL scores between the trans participants and cisgender comparison groups adjusted for age.

Characteristic	Masculinising GAHT Mean (SD)	*n*	Cisgender Females Mean (SD)	*n*	Adjusted Mean Difference (95% CI)	*p* value	Feminising GAHT Mean (SD)	*n*	Cisgender Males Mean (SD)	*n*	Adjusted Mean Difference (95% CI)	*p* value
Gender Preoccupation and Stability Questionnaire												
0 months	41.4 (7.3)	42	19.9 (3.4)	50			42.1 (7.4)	35	18.4 (3.8)	53		
3 months	36.7 (7.8)	38	19.6 (4.4)	49			38.1 (8.0)	34	18.6 (3.6)	52		
6 months	33.8 (8.1)	36	19.1 (3.4)	46	-6.80 (-8.68, -4.91)	**<0.001**	38.5 (8.4)	30	18.3 (3.6)	42	-4.22 (-6.21, -2.24)	**<0.001**
Physical Functioning Score												
0 months	62.6 (18.1)	42	78.8 (13.6)	50			59.2 (19.1)	35	81.8 (10.8)	53		
3 months	64.8 (15.2)	38	75.6 (16.2)	49			61.2 (20.0)	34	80.8 (12.2)	52		
6 months	65.8 (15.3)	36	75.5 (15.1)	46	8.10 (-2.47, 18.67)	0.133	58.1 (20.9)	30	82.8 (11.0)	42	-7.01 (-17.57, 3.55)	0.192
Role limitations due to physical health												
0 months	46.0 (38.9)	42	89.1 (24.4)	50			48.0 (39.5)	35	91.3 (22.1)	53		
3 months	54.8 (38.1)	38	81.8 (31.7)	49			62.2 (41.7)	34	87.4 (26.9)	52		
6 months	54.3 (36.0)	36	88.6 (29.3)	46	-0.67 (-13.03, 11.69)	0.915	50.6 (45.6)	30	94.9 (14.7)	42	-0.21 (-12.72, 12.30)	0.974
Role limitations due to emotional problems												
0 months	44.9 (19.1)	42	61.2 (21.9)	50			45.9 (16.3)	35	65.7 (18.1)	53		
3 months	48.7 (18.1)	38	62.4 (20.0)	49			46.8 (19.4)	34	65.1 (20.9)	52		
6 months	48.1 (18.2)	36	58.8 (22.2)	46	9.21 (-7.53, 25.96)	0.280	47.8 (18.4)	30	66.7 (18.7)	42	-1.83 (-18.93, 15.28)	0.834
Energy/Fatigue												
0 months	61.7 (20.2)	42	80.3 (15.0)	50			66.3 (15.9)	35	77.6 (16.4)	53		
3 months	67.1 (17.2)	38	81.4 (15.1)	49			67.3 (16.9)	34	72.6 (18.3)	52		
6 months	66.5 (21.0)	36	78.9 (16.9)	46	6.27 (-0.81, 13.35)	0.082	65.4 (15.4)	30	72.1 (19.1)	42	1.93 (-5.24, 9.10)	0.597
Emotional Well-Being												
0 months	77.6 (25.5)	42	87.0 (15.7)	50			88.0 (15.1)	35	87.3 (15.0)	53		
3 months	78.7 (23.9)	38	86.4 (15.6)	49			87.8 (12.7)	34	88.4 (15.0)	52		
6 months	80.8 (23.0)	36	86.1 (15.0)	46	7.48 (1.32, 13.64)	**0.018**	83.6 (11.9)	30	86.3 (15.1)	42	-2.54 (-8.76, 3.67)	0.421
Social Functioning												
0 months	84.4 (23.9)	42	97.0 (8.1)	50			89.9 (20.9)	35	89.3 (25.0)	53		
3 months	84.2 (23.4)	38	93.6 (17.6)	49			92.2 (11.3)	34	96.1 (11.4)	52		
6 months	88.7 (18.7)	36	93.2 (20.7)	46	12.50 (2.84, 22.15)	**0.011**	90.0 (17.2)	30	96.8 (7.3)	42	-7.14 (-16.90, 2.62)	0.151
Bodily Pain score												
0 months	81.0 (35.7)	42	95.7 (15.2)	50			95.0 (13.3)	35	95.0 (15.2)	53		
3 months	85.8 (32.6)	38	92.6 (21.3)	49			89.2 (23.4)	34	90.6 (24.6)	52		
6 months	81.5 (35.8)	36	96.6 (13.4)	46	4.83 (-2.37, 12.02)	0.188	90.7 (22.1)	30	90.9 (23.2)	42	-4.01 (-11.20, 3.17)	0.272
General Health score												
0 months	64.6 (22.9)	42	91.6 (13.2)	50			72.5 (23.0)	35	93.5 (11.9)	53		
3 months	72.1 (23.8)	38	87.8 (19.5)	49			71.7 (19.9)	34	90.6 (17.1)	52		
6 months	72.2 (26.0)	36	87.2 (20.1)	46	4.09 (-1.43, 9.62)	0.146	66.2 (29.0)	30	95.1 (9.3)	42	2.40 (-3.14, 7.94)	0.395

Mean differences are adjusted for the age of the participant and all participants at baseline were used in the linear mixed effects model, in which missing data were handled via maximum likelihood estimation. Gender dysphoria was measured by the Gender Preoccupation and Stability Questionnaire, with lower scores indicating less gender dysphoria. The remaining domains are part of the RAND SF36 Health Survey whereby higher scores indicate better QoL.

Bold indicates statistically significant finding.

### Quality of Life

As shown in **Table 2**, individuals initiating masculinising GAHT showed a significant improvement in emotional well-being (mental health) [adjusted mean difference of 7.48 (1.32, 13.64), *p* = 0.018] as well as social functioning aspects of QoL [adjusted mean difference 12.50 (2.84, 22.15), *p* = 0.011] relative to cisgender female comparison group over the first 6 months of treatment. Improvements in both aspects were greater than the minimum clinically important difference (32). No significant differences were observed in the remaining 6 domains: physical functioning, role limitations due to physical functioning, role limitations due to emotional problems, energy/fatigue, bodily pain or general health score.

Individuals initiating feminising GAHT showed no significant changes in QoL over the first 6 months relative to the cisgender male group (**Table 2**).

## Discussion

We demonstrate in this prospective controlled study that in trans people initiating masculinising GAHT, that there is an improvement in gender dysphoria and a clinically significant improvement in emotional well-being and social functioning aspects of QoL over the first 6 months of treatment relative to cisgender female comparison group. In trans people initiating feminising GAHT, there is an improvement in gender dysphoria, but no differences in QoL as measured by the RAND SF-36 were observed.

### Gender Dysphoria

The beneficial effects of GAHT on gender dysphoria have previously been discussed in relation to changes in psychological state, such as changes in body uneasiness (16, 17), social anxiety (6), and self-esteem (19), however specific changes in gender dysphoria have rarely been quantified. To our knowledge, this is the only longitudinal controlled analysis of gender dysphoria, and our findings of improved dysphoria in both the masculinising-hormone and feminising hormone groups relative to the cisgender comparison group over 6 months, are consistent with 2 previous longitudinal but uncontrolled studies which reported lower levels of gender dysphoria after starting GAHT as measured by the Utrecht Gender Dysphoria Scale (23) or the Gender Identity/Gender Dysphoria questionnaire (11). Notably, whilst our findings were statistically significant, the degree of change (4.2 – 6.8 points) is less than the minimum clinically significant difference of 11 points for GPSQ. This is likely related to the limited duration of 6 months follow up which may be insufficient to have a significant effect on an individual’s physical characteristics and in turn, their gender dysphoria. Moreover, contributors to gender dysphoria are complex and GAHT alone may not impact on many other confounders such as social gender role recognition, exposure to discrimination and physical features unaffected by GAHT such as genitalia.

### Quality of Life

We have demonstrated improved emotional well-being and social functioning aspects of QoL in trans people initiating masculinising GAHT over 6 months compared with cisgender females. Whilst no other controlled prospective studies have been performed, our findings are consistent with an Italian cohort study which followed trans men over the first 12 months of masculinising GAHT which showed improved QoL related to body image (22). Moreover, several cross-sectional studies in trans men which have all shown better QoL as measured by SF-36 in those using GAHT compared to those not on GAHT (20, 21, 33, 34). Such consistently positive changes in multiple studies may be reflective of the effectiveness of testosterone therapy in inducing masculinising physical characteristics, and significantly reducing dysphoria, to a greater magnitude than those initiating feminising hormone therapy.

In contrast, we did not demonstrate a significant improvement in QoL in trans women commencing feminising GAHT relative to the cisgender comparison group. Multiple cross-sectional studies examining QoL measured by SF-36 have been performed comparing trans women on GAHT compared with trans women not using GAHT. These have shown variable results with some studies finding worse QoL on GAHT (35), better (20, 34, 36) or no change (30, 37). Whilst the SF-36 is widely used, it is unclear which measure is most appropriate in trans women. Utilising the WHO QoL questionnaire, *Manieri et al.* did find improved overall QoL in trans women over the first 12 months (22). Notably our relatively shorter follow-up over 6 months may be insufficient for feminising GAHT to have maximal effect, and whilst dysphoria significantly improved, many physical characteristics, such as voice pitch and bony structure, which may contribute to social functioning or gender role recognition are unchanged with GAHT alone and multidisciplinary support of gender transition (i.e., with speech pathology, surgery) for trans people seeking feminisation may be needed.

### Limitations

There are multiple limitations to our study. The short follow-up time of 6 months is likely insufficient to gain a complete and thorough understanding of GAHT’s psychological effects, however we were interested in short-term effects of GAHT. Furthermore, we did not recruit a comparison group who identified as trans. A controlled trial in trans people randomised to GAHT or no GAHT would allow the best understanding of GAHT’s effect on gender dysphoria and QoL, however the conduction of such a trial is considered by many trans community members to be unethical, given the existing difficulties in accessing healthcare experienced by many who desire GAHT. As such, a cisgender comparison group were used. We used the locally developed GPSQ to measure gender dysphoria, although this has not been validated as a tool to measure changes in dysphoria over time.

However, this is the only prospective controlled study examining gender dysphoria and QoL in trans individuals newly commencing GAHT, thus validation from future studies and longer term follow up is required.

## Conclusions

In the short-term, our findings support the benefit of initiating feminising or masculinising GAHT on gender dysphoria. Masculinising GAHT improves emotional well-being and social functioning within 6 months of treatment however there are many other confounders which can contribute to gender dysphoria or QoL other than the changes in physical characteristics induced by GAHT. These confounders include the presence of dysphoria towards one’s voice, chest or genitalia which may not be relieved with GAHT, the presence of a supportive home, school or work environment, relationship status or sexual function. Multidisciplinary input to holistically support trans people, particularly those seeking feminisation will likely be of benefit. Further longitudinal studies controlled for confounders contributing to QoL will provide greater insights on the benefit of GAHT specifically on gender dysphoria.

## Data Availability Statement

The raw data supporting the conclusions of this article will be made available by the authors, without undue reservation.

## Ethics Statement

The study was approved by the Austin Health Human Research and Ethics Committee (HREC/17/Austin/74). The patients/participants provided their written informed consent to participate in this study.

## Author Contributions

Conceptualization, IB, JDZ, and AC. Methodology, LF, IB, JZ, and AC. Investigation, LF, IB, and SL. Formal analysis, LF, IB, SL, and AC. Writing – original draft, LF and AC. Writing – review and editing, LF, IB, SL, JZ, and AC. Funding acquisition, AC. Supervision, AC. All authors contributed to the article and approved the submitted version.

## Funding

AC is supported by a National Health and Medical Research Council of Australia Early Career Fellowship (Grant number: 1143333). This study was supported by funding received from Austin Medical Research Foundation, Endocrine Society of Australia, RACP Foundation and Viertel Charitable Foundation. IB is supported by an Australian Postgraduate Award.

## Conflict of Interest

The authors declare that the research was conducted in the absence of any commercial or financial relationships that could be construed as a potential conflict of interest.

## Publisher’s Note

All claims expressed in this article are solely those of the authors and do not necessarily represent those of their affiliated organizations, or those of the publisher, the editors and the reviewers. Any product that may be evaluated in this article, or claim that may be made by its manufacturer, is not guaranteed or endorsed by the publisher.
